# Application of SARIMA model in forecasting and analyzing inpatient cases of acute mountain sickness

**DOI:** 10.1186/s12889-023-14994-4

**Published:** 2023-01-09

**Authors:** Jianchao Liu, Fangfang Yu, Han Song

**Affiliations:** 1 Institute of Hospital Management, Department of Medical Innovation Research, PLA General Hospital, Beijing, 100853 China; 2 Department of Medical Innovation Research, PLA General Hospital, Beijing, 100853 China; 3 Department of Health Service, PLA General Hospital, No.28, Fuxing Road, Beijing, 100853 China

**Keywords:** Acute Mountain Sickness, Time series, Seasonal Auto-Regressive Integrated Moving Average (SARIMA), Prediction

## Abstract

**Background:**

Acute Mountain Sickness (AMS) is typically triggered by hypoxia under high altitude conditions. Currently, rule of time among AMS inpatients was not clear. Thus, this study aimed to analyze the time distribution of AMS inpatients in the past ten years and construct a prediction model of AMS hospitalized cases.

**Methods:**

We retrospectively collected medical records of AMS inpatients admitted to the military hospitals from January 2009 to December 2018 and analyzed the time series characteristics. Seasonal Auto-Regressive Integrated Moving Average (SARIMA) was established through training data to finally forecast in the test data set.

**Results:**

A total of 22 663 inpatients were included in this study and recorded monthly, with predominant peak annually, early spring (March) and mid-to-late summer (July to August), respectively. Using the training data from January 2009 to December 2017, the model SARIMA (1, 1, 1) (1, 0, 1) 12 was employed to predict the test data from January 2018 to December 2018. In 2018, the total predicted value after adjustment was 9.24%, less than the actual value.

**Conclusion:**

AMS inpatients have obvious periodicity and seasonality. The SARIMA model has good fitting ability and high short-term prediction accuracy. It can help explore the characteristics of AMS disease and provide decision-making basis for allocation of relevant medical resources for AMS inpatients.

## Introduction

Acute mountain sickness (AMS) occurs when people arrive in an area 2500 m above sea level, and fail to adapt to high altitude in physiological aspects. It is characterized by headache, insomnia, dizziness, fatigue, and gastrointestinal reactions (anorexia, nausea or vomiting) and other symptoms [1, 2]. The plateaus in China are mainly distributed in regions including Xinjiang, Tibet, Qinghai, etc., as well as parts of high lands of Yunnan, Gansu, and Sichuan. The plateau has a complex geographic environment, with high altitude, low oxygen partial pressure, cold climate, heavy and dry winds, and frequent natural disasters [3]. Construction workers, residents, and disaster reliefs and poverty alleviation teams in Tibet all have reported various degrees of AMS. Among the builders of the Qinghai-Tibet Railway, the incidence of AMS due to the first high altitude exposure was more than 50% [4]. After entering Tibet from the plains, 146 out of 640 passengers presented with AMS at an incidence rate of about 23% [5]. After the “4·14” earthquake in Yushu, Qinghai, 193 officers and workers flew to the plateau for rescue and relief. Among them, 154 cases reported AMS, with a high incidence rate close to 80% because of relatively heavy physical exertion [6].

In the past 10 years, a large number of studies focused on the incidence of AMS after high-altitude exposure under various conditions, but the admission of AMS inpatients remained unclear. This study aimed to analyze and predict the time series of AMS inpatients admitted to military hospitals in plateau areas in the past 10 years by means of the Seasonal Auto-Regressive Integrated Moving Average (SARIMA). The trend may provide a reference for allocation of related medical resources used for treatment of the diseases.

## Materials and methods

### Data collection

The medical records were collected, with information related to AMS inpatients admitted to military hospital from January 2009 to December 2018. The hospitals in plateaus served both military personnel and civilians. The AMS standardized diagnosis (T70.2) under the International Disease Classification System ICD-10 were used as the retrieval basis. The train set and test set constituted 95.18%, 4.82% of the total data, respectively. The present study was performed with historical de-identified data; thus, it was exempt from Institutional Review Board approval.

### Model establishment

The statistical modeling of AMS inpatients was analyzed by Statistical Product Service Solutions (SPSS) 22.0, specifically following three steps: variance analysis, model building and predictive analysis. Variance analysis incorporated normality test, overall comparison analysis between groups, and pairwise comparison analysis. Model building focused on the stationarity discrimination, model selection, white noise test, and parameter training. Predictive analysis was carried out to calculate monthly admissions based on the prediction model.

#### Variance analysis

A frequency distribution histogram and QQ diagram of monthly AMS admissions were charted. The normality was evaluated by Shapiro–Wilk (S-W) test. The differences of monthly admissions were tested using one-way ANOVA. The Levene’s statistic test was utilized for assumption of homogeneity of variances. The Dunnett T3 method was employed for further pairwise comparisons between any two groups if the variances unequal.

#### Model selection

ARIMA (*p, d, q*) or ARMA (*p, q*) was combination of autoregressive (AR) and moving average (MA) models with or without differencing. AR (p) was presented in the equation below, where ${y}_{t}, {y}_{t-1},{y}_{t-2},{y}_{t-p}$ are stationaries, and ${\phi }_{0},{\phi }_{1},{\phi }_{2},{\phi }_{p}$ are constants. ${\varepsilon }_{\mathrm{t}}$ is a Gaussian white noise series with mean zero.

$${y}_{t}={{\phi }_{0}+{\phi }_{1}{y}_{t-1}+{\phi }_{2}{y}_{t-2}+\dots +{\phi }_{p}{y}_{t-p}+\varepsilon }_{\mathrm{t}}$$

MA (q) was presented in the equation below, where there are q lags in the moving average and ${\theta }_{1},{\theta }_{2},{\theta }_{q}$ are parameters. ${\varepsilon }_{\mathrm{t}},{\varepsilon }_{\mathrm{t}-1},{\varepsilon }_{\mathrm{t}-2},{\varepsilon }_{\mathrm{t}-\mathrm{q}}$ is a Gaussian white noise series with mean zero.

$${y}_{t}={\varepsilon }_{\mathrm{t}}-{\theta }_{1}{\varepsilon }_{t-1}-{\theta }_{2}{\varepsilon }_{t-2}-\dots -{\theta }_{q}{\varepsilon }_{t-q}$$

Here, backshift operator (B) was introduced as described below.

$${B}^{n}{y}_{t}={y}_{t-n}$$

Thus, ARIMA (*p, d, q*) was presented briefly in the equation below, where ∇^d^ is the difference operator:${(1-B)}^{d}$, $\Theta (B)$ is the moving average polynomial:$1-{\theta }_{1}B-\dots -{\theta }_{q}{B}^{q}$, $\Phi (B)$ is an autoregressive polynomial:$1-{\phi }_{1}B-\dots -{\phi }_{p}{B}^{p}$.

$${\Phi (B)\nabla }^{\mathrm{d}}{y}_{t}={\Theta (B)\varepsilon }_{\mathrm{t}}$$

The SARIMA model based on non-seasonal model ARIMA (*p, d, q*) and seasonal model ARIMA (*P, D, Q*)*S* was employed for prediction of seasonal, nonstationary time series. The model, denoted generally as ARIMA (*p, d, q*) × ARIMA(*P, D, Q*)*S*, was presented in the equation below, where ∇^d^ is the difference operator:${(1-B)}^{d}$, ${\nabla }_{S}^{D}$ is the seasonal difference operator: ${(1-{B}^{S})}^{D}$,$\Theta (B)$ is the moving average polynomial:$1-{\theta }_{1}B-\dots -{\theta }_{q}{B}^{q}$, ${\Theta }_{S}(B)$ is the seasonal moving average polynomial:$1-{\theta }_{1}{B}^{S}-\dots -{\theta }_{Q}{B}^{QS}$, $\Phi (B)$ is an autoregressive polynomial:$1-{\phi }_{1}B-\dots -{\phi }_{p}{B}^{p}$, ${\Phi }_{S}(B)$ is an seasonal autoregressive polynomial:$1-{\phi }_{1}{B}^{S}-\dots -{\phi }_{P}{B}^{PS}$.

$${\nabla }^{\mathrm{d}}{\nabla }_{S}^{D}{y}_{t}=\frac{\Theta (B)\times {\Theta }_{S}(B)}{\Phi (B)\times {\Phi }_{S}(B)}{\varepsilon }_{t}$$

##### Stationarity test

Time series of AMS inpatients admissions was established to initially judge the stationarity of the series. Auto Correlation Function (ACF) graphs and Partial Auto Correlation Function (PACF) graphs were drawn. Data differentiation was determined according to the attenuation of autocorrelation coefficients and partial autocorrelation coefficients. Augmented Dickey-Fuller (ADF) unit root test was used to identify the trend and periodicity.

##### Model selection

The appropriate value ranges for *p, d, q* and *P, D, Q*, and *S* were also determined by tailing and truncation of the ACF and PACF graphs.

##### Parameter estimation

The Least Squares were adopted to estimate the parameters, and white noise test was performed for the selected ARIMA(*p, d, q*) × ARIMA(*P, D, Q*)*S* to identify the time series information extraction. Normalized bayesian information criteria (*BIC*) was used to determine the degree of overfitting.

#### Predictive analysis

The data was divided into a training set and a test set. Time span for training data lasted from January 2009 to December 2017 while that for the test data ranged from January to December 2018.

## Result

### Data distribution

**Fig. 1:**
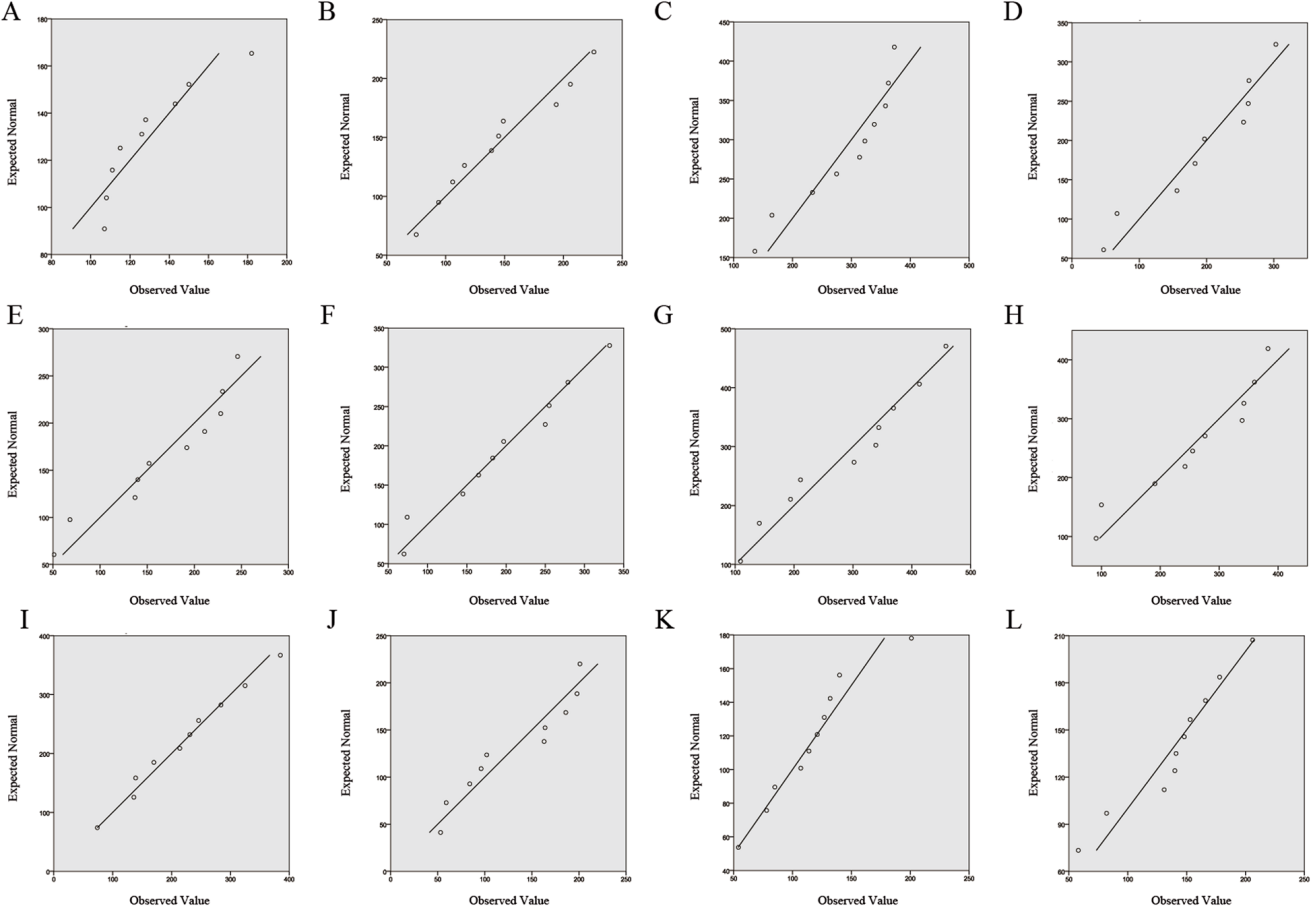
Distribution of AMS inpatients monthly admissions. **A**, **B**, **C**, **D**, **E**, **F**, **G**, **H**, **I**, **J**, **K**, **L** correspond to QQ map of the number of AMS inpatients admitted in January, February, March, April, May, June, July, August, September, October, November, and December

The QQ chart showed that the numbers of AMS inpatients from January to December (Fig. 1), and the S-W test showed that the *W* in statistics were 0.84, 0.95, 0.88, 0.92, 0.91, 0.96, 0.95, 0.91, 0.98, 0.89, 0.95, 0.94, from January to December (*p* > 0.05), indicating that the monthly data obeyed normal distribution (Table 1).

**Table 1:** Normality test (S-W test) of the number of admissions of AMS inpatients

	January	February	March	April	May	June	July	August	September	October	November	December
*W*	0.84	0.95	0.88	0.92	0.91	0.96	0.95	0.91	0.98	0.89	0.95	0.94
*p*	0.05	0.69	0.12	0.37	0.31	0.80	0.66	0.29	0.98	0.16	0.67	0.54

### Analysis of variance

**Fig. 2:**
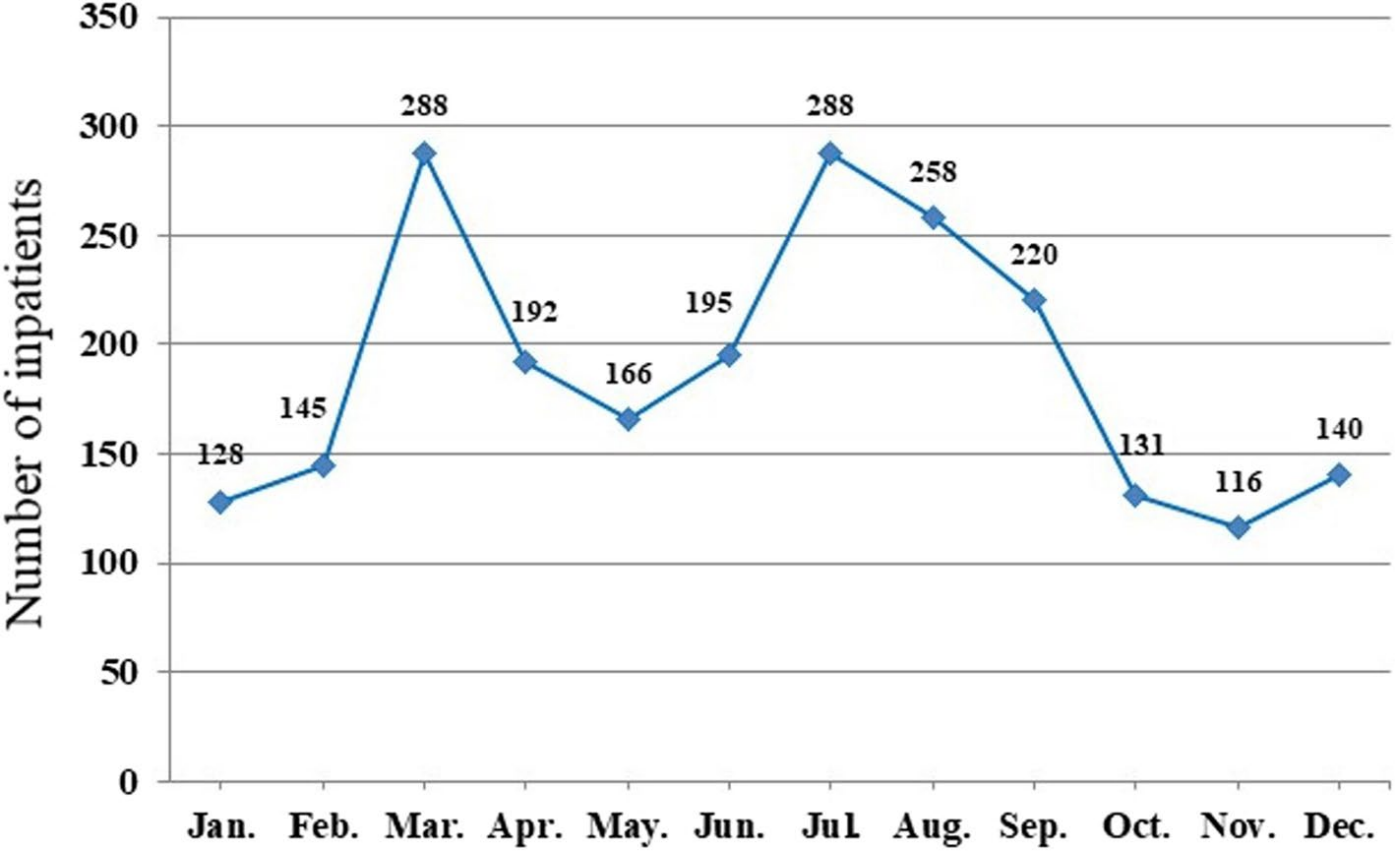
The average monthly admissions of AMS patients from 2009 to 2018

The homogeneity of variance test found unequal variances (*F* = 3.39, *p* < 0.01). One-way ANOVA results showed the significant differences among groups (*F* = 6.67, *p* < 0.01). As illustrated in the line chart, AMS presented in a shape of dual-peaks and triple-dips, with higher levels in early spring and mid-to-late summer, and lower levels in late autumn and winter (Fig. 2). The Dunnett T3 test compares differences between two groups. The number of AMS inpatients reached highest in March and July, which is significantly higher than other months (*p* < 0.05) (Table 2).

**Table 2:** Multiple comparisons of the number of admissions of AMS inpatients from January to December

Month (*I*)	Month (*J*)	Mean difference (*I-J*)	*p*	*LCI*	*UCI*
January	February	-16.90	1.00	-90.01	56.21
	March	-159.90*	0.01	-280.41	-39.39
	April	-63.50	0.72	-184.76	57.76
	May	-37.40	0.98	-135.18	60.38
	June	-66.90	0.67	-189.92	56.12
	July	-159.90*	0.07	-328.99	9.19
	August	-129.80	0.11	-278.96	19.36
	September	-92.30	0.35	-227.91	43.31
	October	-2.50	1.00	-86.10	81.10
	November	12.20	1.00	-47.93	72.33
	December	-12.20	1.00	-76.31	51.91
February	March	-143.00*	0.02	-268.64	-17.36
	April	-46.60	0.99	-172.90	79.70
	May	-20.50	1.00	-126.75	85.75
	June	-50.00	0.99	-177.88	77.88
	July	-143.00	0.15	-313.91	27.91
	August	-112.90	0.28	-264.84	39.04
	September	-75.40	0.76	-214.72	63.92
	October	14.40	1.00	-80.89	109.69
	November	29.10	1.00	-51.24	109.44
	December	4.70	1.00	-77.81	87.21
March	April	96.40	0.55	-51.67	244.47
	May	122.50	0.10	-12.62	257.62
	June	93.00	0.62	-56.18	242.18
	July	0.00	1.00	-182.86	182.86
	August	30.10	1.00	-137.19	197.39
	September	67.60	0.98	-89.92	225.12
	October	157.40*	0.01	28.30	286.50
	November	172.10*	< 0.01	49.52	294.68
	December	147.70*	0.01	24.33	271.07
April	May	26.10	1.00	-109.58	161.78
	June	-3.40	1.00	-153.03	146.23
	July	-96.40	0.84	-279.54	86.74
	August	-66.30	0.99	-233.94	101.34
	September	-28.80	1.00	-186.72	129.12
	October	61.00	0.93	-68.72	190.72
	November	75.70	0.56	-47.58	198.98
	December	51.30	0.97	-72.77	175.37
May	June	-29.50	1.00	-166.51	107.51
	July	-122.50	0.38	-298.08	53.08
	August	-92.40	0.69	-250.57	65.77
	September	-54.90	1.00	-201.82	92.02
	October	34.90	1.00	-76.34	146.14
	November	49.60	0.89	-51.88	151.08
	December	25.20	1.00	-77.57	127.97
June	July	-93.00	0.88	-276.83	90.83
	August	-62.90	1.00	-231.38	105.58
	September	-25.40	1.00	-184.28	133.48
	October	64.40	0.90	-66.80	195.60
	November	79.10	0.51	-45.86	204.06
	December	54.70	0.95	-71.01	180.41
July	August	30.10	1.00	-165.86	226.06
	September	67.60	1.00	-121.64	256.84
	October	157.40	0.09	-15.10	329.90
	November	172.10*	0.05	2.42	341.78
	December	147.70	0.12	-22.28	317.68
August	September	37.50	1.00	-137.49	212.49
	October	127.30	0.17	-26.82	281.42
	November	142.00	0.07	-8.15	292.15
	December	117.60	0.21	-33.00	268.20
September	October	89.80	0.55	-52.23	231.83
	November	104.50	0.24	-32.52	241.52
	December	80.10	0.63	-57.50	217.70
October	November	14.70	1.00	-74.32	103.72
	December	-9.70	1.00	-100.46	81.06
November	December	-24.40	1.00	-97.84	49.04

^*^p < 0.05

### Time series

**Fig. 3:**
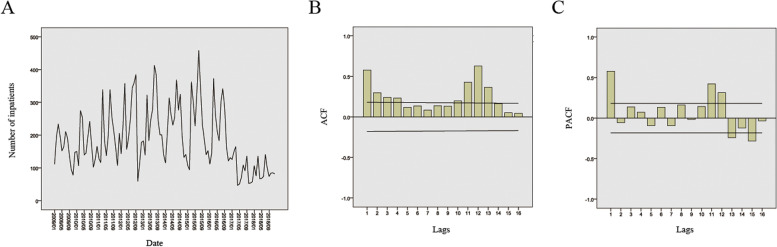
Monthly admissions of AMS inpatients in military hospitals from 2009 to 2018 (**A**) and analysis of autocorrelation (**B**) and partial autocorrelation (**C**)

Time series chart of the monthly admissions of AMS inpatients was drawn. The line graph exhibits the over-time admissions with a seasonal fluctuation in a downward trend (Fig. 3A). The autocorrelation coefficient and partial autocorrelation coefficient did not follow the lag order. The number gradually decreased, showing a tailing trend (Fig. 3B&C). The ADF unit root test showed that the original series was not stationary (*t* = -0.39, *p* = 0.91), which needed first-order difference to become a stationary series (*t* = -6.56, *p* < 0.01).

### Model selection and parameter estimation

The SARIMA model was selected to deal with short-term correlation and seasonal effects. The white noise test statistics Ljung-Box *Q*, Stationary *R*-squared, and the penalty function Normalized *BIC* were used to determine *p, d, q* and *P, D, Q, S*. Without seasonal information in prediction models, Stationary *R*-squared of AR, MA, ARMA, ARIMA were less than 0.5. We found SARIMA (1,1,1) × (1,0,1)12 fully extracted information, with a non-white noise sequence (*Q* = 18.97, *p* = 0.01). The model had largest stability determination coefficient, and lowest function value and relatively better prediction (Table 3).

**Table 3:** Comparison of prediction performance of different parameter

Model	Ljung-Box *Q*	*p*	Stationary *R*-squared	Normalized *BIC*
AR (1)	51.25	< 0.01	0.28	8.88
MA (1)	56.25	< 0.01	0.26	8.91
ARMA (1,1)	46.88	< 0.01	0.29	8.92
ARIMA (1,1,1)	60.29	< 0.01	0.26	8.89
ARIMA (0,1,1)	88.04	< 0.01	0.07	9.06
ARIMA (1,1,0)	71.40	< 0.01	0.03	9.12
SARIMA (1,1,1) × (1,1,1)12	24.10	0.05	0.54	8.50
SARIMA (1,1,1) × (0,1,1)12	20.85	0.14	0.55	8.42
SARIMA (1,1,1) × (1,1,0)12	22.28	0.10	0.49	8.54
SARIMA (1,1,1) × (1,0,0)12	20.01	0.17	0.53	8.49
SARIMA (1,1,1) × (1,0,1)12	18.97	0.17	0.59	8.41

The parameter estimation displays statistically significant constant and coefficient, of which constant term was 148.48, autoregressive term coefficient was 0.47, moving average term coefficient was 1.00, seasonal autoregressive term coefficient was 1.00, and seasonal moving average term coefficient was 0.93 (Table 4).

**Table 4:** SARIMA model parameter estimation and significance test results

parameter	estimate	standard error	*t*	*p*
constant	148.48	37.30	3.98	< 0.01
AR (1)	0.47	0.09	5.11	< 0.01
MA (1)	1.00	0.11	8.97	< 0.01
AR (1), seasonal	1.00	0.03	36.70	< 0.01
MA (1), seasonal	0.93	0.45	2.06	0.04

### Model forecast

**Fig. 4:**
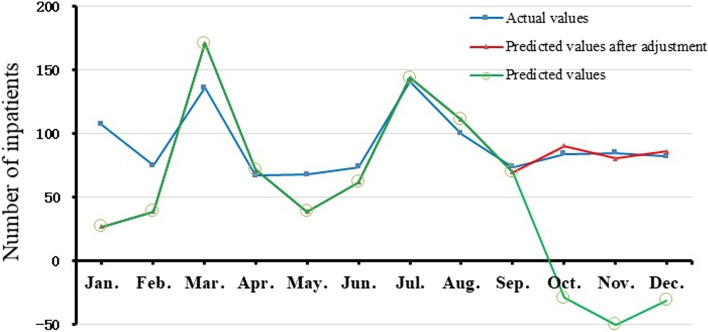
Prediction of admissions of AMS inpatients from January to December 2018

The forecast results show that the forecast value from January to September was generally consistent with actual ones. In 2018, we totally predicted 625 AMS hospitalized inpatients under the SARIMA (1,1,1) × (1,0,1)12 model. However, given that the forecast was negative for October (-29), November (-50), and December (-31). These data were replaced by the average value of its’ respective previous two months. Finally, after adjustment, 992 AMS hospitalized inpatients were calculated, whereas the number of actual inpatients was 1093 (Fig. 4).

## Discussion

AMS is a syndrome that contains a variety of non-specific symptoms. It is highly subjective and occurs frequently in people who rapidly enter high-altitude areas from plain places, primarily in the case of military training, construction in Tibet, tourist mountaineering groups. The onset of AMS is closely related to various factors such as altitude, speed, age, or gender. However, there was a lack of investigation and prediction on AMS populations’ time series. Constant with previous studies, we found obvious seasonality and cyclicality of AMS inpatients [7].

As a classic time series model, SARIMA effectively captured periodic and seasonal changes, especially for regular pattern of diseases [8]. In this study, the SARIMA (1, 1, 1) (1, 0, 1) 12 model was used to fit the number of AMS hospitalized cases from 2009 to 2018. The total predicted value was 9.24% less than actual one, showing relatively precise predictive abilities.

Although plateau climate becomes warmer in March, the temperature gap between day and night was still large, and the adverse impact on the body may not be overcome [9]. In the spring of 2002, the US military carried out the "Operation Python" in the mountainous area at an altitude between 610 and 3,600 m, reporting a death toll of 8 people and 80 injuries, of which 10 suffered from AMS. The reduction rate derived from AMS accounted for 11.36%. Thus, altitude adaptation and medical training were further strengthened in US troops [10]. The high incidence in July may be closely related to seasonal tourism, where the scenery of the plateau region was favored by lots of tourists. Travellings from the mainland to high-altitude scenic spots were found from June to August almost every year. The incidence of AMS during this period was about 64%, considering most travelers quickly entering the plateau and lack pre-adaptation to the low-oxygen environment [11].

This study has some limitations. First, we used the information of medical records of AMS hospitalized inpatients. The trend presented by this time series only reflected the current situation, but restricted to evaluate the severity of the disease. The sharp increase in AMS admissions in March and July may be related to large number of people going to the plateau [7]. Second, SARIMA’s short- and medium-term forecasting showed a better performance than the long-term. The forecasted values from January to September were more consistent with the actual ones. We adjusted the forecasted values of October, November, and December by using the average value of its’ previous two months, respectively. So, researchers may be cautious to the longer-term results. Third, we did not consider factors such as the altitude and speed of entering the plateau, and history of altitude sickness. To make better use model prediction, various factors that affect AMS should be incorporated within comprehensive analysis.

In summary, hospitalized inpatients with AMS showed obvious periodicity and seasonality. The SARIMA model has sound fitting and high short-term prediction accuracy. This study may be helpful for investigating the general characteristics of AMS inpatients, facilitating the allocation of relevant medical resources.

## Data Availability

The data that support the findings of this study are available from Directorate of Medical Services, Logistics Support Department of PLA, but restrictions apply to the availability of these data, which were used under license for the current study, and so are not publicly available. Data are however available from the authors upon reasonable request and with permission of Directorate of Medical Services, Logistics Support Department of PLA.
